# Benefits of peritoneal ultrafiltration in HFpEF and HFrEF patients

**DOI:** 10.1186/s12882-020-01777-x

**Published:** 2020-05-14

**Authors:** Leonie Grossekettler, Bastian Schmack, Carsten Brockmann, Reinhard Wanninger, Michael M. Kreusser, Lutz Frankenstein, Lars P. Kihm, Martin Zeier, Hugo A. Katus, Vedat Schwenger, Andrew Remppis

**Affiliations:** 1 Department of Internal Medicine III, Cardiology, Angiology and Pulmonology, University Hospital of Heidelberg, Im Neuenheimer Feld 410, 69120 Heidelberg, Germany; 2 Clinic for Cardiac Surgery, University Hospital of Heidelberg, Heidelberg, Germany; 3 Clinic for Cardiology, Heart and Vascular Center, Bad Bevensen, Germany; 4 Department of Nephrology, Medical Center, Bad Bevensen, Germany; 5 Department of Nephrology, Clinic Braunschweig, Braunschweig, Germany; 6 Department of Internal Medicine I, Endocrinology and Nephrology, University Hospital of Heidelberg, Heidelberg, Germany; 7 Department of Kidney-, Blood Pressure- and Autoimmune Diseases, Katharinenhospital, Klinikum Stuttgart, Stuttgart, Germany

**Keywords:** Heart failure, HFpEF, HFrEF, Cardiorenal syndrome, Peritoneal dialysis, Ultrafiltration

## Abstract

**Background:**

Peritoneal ultrafiltration (pUF) in refractory heart failure (HF) reduces the incidence of decompensation episodes, which is of particular significance as each episode incrementally adds to mortality. Nevertheless, there are insufficient data about which patient cohort benefits the most. The objective of this study was to compare pUF in HFrEF and HFpEF, focusing on functional status, hospitalizations, surrogate endpoints and mortality.

**Methods:**

This study involves 143 patients, who could be classified as either HFpEF (*n* = 37, 25.9%) or HFrEF (*n* = 106, 74.1%) and who received pUF due to refractory HF.

**Results:**

Baseline eGFR was similar in HFrEF (23.1 ± 10.6 mg/dl) and HFpEF (27.8 ± 13.2 mg/dl). Significant improvements in NYHA class were found in HFpEF (3.19 ± 0.61 to 2.72 ± 0.58, *P* <  0.001) and HFrEF (3.45 ± 0.52 to 2.71 ± 0.72, *P* <  0.001). CRP decreased in HFrEF (19.4 ± 17.6 mg/l to 13.7 ± 21.4 mg/l, *P* = 0.018) and HFpEF (33.7 ± 52.6 mg/l to 17.1 ± 26.3 mg/l, *P* = 0.004). Body weight was significantly reduced in HFrEF (81.1 ± 14.6 kg to 77.2 ± 15.6 kg, *P* = 0.003) and HFpEF (86.9 ± 15.8 kg to 83.1 ± 15.9 kg, *P* = 0.005). LVEF improved only in HFrEF (25.9 ± 6.82% to 30.4 ± 12.2%, *P* = 0.046). BCR decreased significantly in HFrEF and HFpEF (55.7 ± 21.9 to 34.3 ± 17.9 *P* > 0.001 and 50.5 ± 68.9 to 37.6 ± 21.9, *P* = 0.006). Number of hospitalization episodes as well as number of hospitalization days decreased significantly only in HFpEF (total number 2.88 ± 1.62 to 1.25 ± 1.45, *P* <  0.001, days 40.4 ± 31.7 to 18.3 ± 22.5 days, *P* = 0.005).

**Conclusions:**

pUF offers various benefits in HFpEF and HFrEF, but there are also substantial differences. In particular, hospitalization rates were found to be significantly reduced in HFpEF patients, indicating a greater medical and economical advantage. However, LVEF was only found to be improved in HFrEF patients. While pUF can now be regarded as an option to supplement classical HF therapy, further studies are desirable to obtain specifications about pUF in HFpEF, HFmEF and HFrEF patients.

## Background

The global prevalence of heart failure (HF) is increasing, due to ageing populations, insufficiently controlled cardiovascular risk factors as well as prolonged survival in consequence of evidence-based treatments [1]. The rising frequency of HF leads to growing problems – medically, economically and ethically.

Especially in patients over 60 years, HF is the main reason for hospitalization [2]. In the US, 30 day all cause readmission rate is 19%. In Europe 1-year rehospitalization rates run up to 44%, while 32% of outpatients experience a first hospitalization [3].

HF can be classified as heart failure with reduced left ventricular ejection fraction (HFrEF) (LVEF < 40%), heart failure with preserved ejection fraction (HFpEF) and recently heart failure with mid-range ejection fraction (HFmEF, LVEF 40–55%). Prognosis of HF is known to be rather poor: Overall 1-year mortality is 8.8% in HFrEF and 6.3% in HFpEF [4]. HF index admission mortality is around 10% with a post-discharge 30-day and 1-year mortality of 6.5 and 30%, respectively [5]. It has to be noted, that HFrEF therapy is based on a wide range of studies yielding a sound basis for an evidence-based medical approach [6, 7], while HFpEF therapy is largely devoid of any scientific evidence.

The cardiorenal syndrome is an overarching pathophysiology in HF, irrespective of EF [1, 8–11]. It is associated with worse outcome with more than 40% of all-cause mortality being attributable to this co-morbid situation and which represents the main driver for recurrent hospitalizations [12–15].

Due to critical changes in intraglomerular filtration pressures, renal venous congestion and arterial underfilling both lead to “excretory renal insufficiency” with an inadequate volume control triggering recurring hydropic decompensations [10, 14–21]. Importantly, neurohormonal imbalance in HF contributes to these hydropic decompensations by hindering adequate excretion of sodium and water. Therefore, loop diuretics are recommended in current guidelines as first line therapy in patients with acute and chronic decompensated HF (ESC, AHA, ACCF) [6, 7]. Main focus is symptom relief and co-morbidity therapy, suggesting UF or haemofiltration as possible treatment option but fail to specify any details [22]. However, until now no evidence has emerged on superiority of extracorporeal UF compared to diuretic strategies [23–28].

Peritoneal UF (pUF) in patients with in end stage HF and refractory congestion can offer an additional treatment option [29] and is associated with improved New York Heart Association (NYHA) classification and reduced hospitalizations, as we could demonstrate in our previous study [30]. Although frequently used and suggested by the German Societies of Cardiology and Nephrology for treatment of patients with chronic refractory cardiorenal syndrome [31], no data are available whether outcome in HF patients treated with pUF differs with respect to the underlying cardiac pathophysiology and which patient cohort would clinically benefit the most from pUF.

Therefore, the aim of this study was to determine possible advantages and disadvantages of pUF treatment in HFpEF versus HFrEF (according to ESC 2012) [1].

## Methods

This is a substudy of our prospective observational multicentre study, based on the national registry data of the *German Society of Nephrology (DGfN),* to evaluate the efficacy of pUF in patients with refractory HF, this time focusing on HFpEF and HFrEF. One hundred forty-three patients with symptomatic end-stage CHF, classified as either HFpEF or HFrEF, were enrolled in ambulatory pUF therapy after interdisciplinary assessment. Enrolment period took place between January 2010 and December 2014. The inclusion criteria are listed below:

- Individually optimized pharmacological therapy according to the recommendation of the *European Society of Cardiology (ESC)* [1]
- Diuretic resistance defined as refractory hypervolemia despite optimal sequential diuretic therapy (loop diuretics, thiazides and, if possible, mineralocorticoid receptor antagonists (MRA) as recommended by national authorities [1, 28]
- Device therapy as indicated by current guidelines [1]
- Recurrent hospitalizations due to cardiac decompensation, at least 2x within the last 6 months
- Patients not eligible for heart transplantation

Exclusion criteria for this study were defined as standard contraindications for pUF as well as inotropic support [28]. The registry did not include any acute peritoneal dialysis (PD) initiations on ICU. The Modification of Diet in Renal Disease (MDRD) equation was used to calculate renal function, as estimated by glomerular filtration rate (eGFR).

Before confirming the indication for pUF, patients received optimization of conservative medical HF therapy and echocardiography to determine LVEF. It was sought to formerly exclude diagnosis of specific renal pathologies e.g. glomerulonephritis.

Patients were carefully instructed after implantation of a peritoneal dialysis catheter. pUF modalities were continuous ambulatory PD (CAPD), automated PD (APD) or intermittent PD (IPD). Study visits were scheduled at initiation of pUF, at 3 and 6 months, and subsequently in 6 months periods. Primary end-point of the study was defined as all-cause hospitalizations.

All patients provided written informed consent prior to study enrolment. The study was approved by the Ethics Committee of the Medical Faculty of Heidelberg (reference number S-106/2011).

Statistical analysis were performed using IBM SPSS Statistics 24 and Microsoft Excel 2011, including Kolmogorov-Smirnov test, Wilcoxon signed-rank test or Students t-test for paired variables, Levene’s test, Pearson’s correlation, as well as Kaplan-Meier estimator and log-rank test (level of significance α = 5%).

## Results

The study population included 143 patients with a median follow-up time of 302 days (range 9 to 2357). According to ESC 2012 [1], 106 patients were classified as HFrEF (EF < 40%) (74.1%) and 37 patients were classified as HFpEF (EF > 40%) (25.9%). Patient characteristics and treatment modalities of both study groups are demonstrated in Table 1.

**Table 1:** Baseline patient characteristics. Medical and demographic data. Data are presented as mean ± standard deviation (SD) or number

	n	(Percent)
	143	(100%)
**Characteristic of HF**		
HF with reduced EF (< 40%)	106	(74.1%)
HF with preserved EF (> 40%)	37	(25.9%)
**Sex** (male: female)		
Total	m 119: f 24	(83.2%: 16.8%)
HFrEF^b^	m 91: f 15	(63.6%: 10.5%)
HFpEF^c^	m 28: f 9	(19.5%: 6.29%)
**Etiology of CHF**^**a**^		
HFrEF^b^		
Ischemic cardiomyopathy	48	(33.6%)
Dilated cardiomyopathy	36	(25.2%)
Pulmonary hypertension and right ventricular dysfunction	2	(1.40%)
Hypertensive heart disease	1	(0.70%)
Congenital heart defect	1	(0.70%)
Not specified	18	(12.6%)
HFpEF^c^		
Ischemic cardiomyopathy	7	(4.90%)
Dilated cardiomyopathy	8	(5.59%)
Pulmonary hypertension and right ventricular dysfunction	6	(4.20%)
Pericarditis constrictiva	2	(1.40%)
Hypertensive heart disease	1	(0.70%)
Congenital heart defect	1	(0.70%)
Not specified	12	(8.39%)
**Valvular heart disease**		
HFrEF^b^		
Tricuspid regurgitation I°	11	(7.69%)
II°	22	(15.4%)
III°	9	(6.29%)
Mitral regurgitation I°	14	(9.79%)
II°	27	(18.9%)
III°	8	(5.59%)
HFpEF^c^		
Tricuspid regurgitation I°	1	(0.70%)
II°	5	(3.50%)
III°	7	(4.90%)
Mitral regurgitation I°	3	(2.10%)
II°	5	(3.50%)
III°	2	(1.40%)
**Medication**		
HFrEF ^b^		
Angiotensin Converting Enzyme Inhibitors / Angiotensin Receptor Blockers	58	(40.6%)
Beta Blockers	69	(48.3%)
Spironolacton	40	(28.0%)
HFpEF^c^		
Angiotensin Converting Enzyme Inhibitors / Angiotensin Receptor Blockers	20	(14.0%)
Beta Blockers	21	(14.7%)
Spironolacton	13	(9.09%)
Erythropoietin	8	(5.59%)
**pUF**^d^**regime at beginning**		
HFrEF^b^		
APD^e^	36	(25.2%)
CAPD^f^	54	(37.8%)
IPD^g^	3	(2.10%)
HFpEF^c^		
APD^e^	14	(9.79%)
CAPD^f^	18	(12.6%)
IPD^g^	1	(0.70%)
**Haemodialysis prior to pUF**^d^		
HFrEF^b^	11	(7.69%)
HFpEF^c^	5	(3.50%)

^a^CHF (congestive heart failure)

^b^HFrEF (heart failure with reduced ejection fraction)

^c^HFpEF (heart failure with preserved ejection fraction)

^d^pUF (peritoneal ultrafiltration)

^e^APD (automatic peritoneal dialysis)

^f^CAPD (continuous ambulatory peritoneal dialysis)

^g^ IPD (intermittent peritoneal dialysis)

Pre-pUF, 51 HFrEF patients (47.7%) and 20 HFpEF patients (54.1%) received cardiac catheterization. In 57 HFrEF patients (53.3%) and 9 HFpEF patients (23.3%) an implantable cardioverter-defibrillator (ICD) was implanted. 11 HFrEF patients (10.3%) and 5 HFpEF patients (13.5%) required precursory intermittent haemodialysis before starting pUF, main reasons were acute hypervolemia or hyperkalaemia (in HFrEF mean duration of haemodialysis 11.9 ± 1.96 h per week, or 3 times a week with average period of 3.97 ± 0.65 h, respectively; average blood flow 235.0 ± 24.2 ml/min, in HFpEF mean duration of haemodialysis 14.8 ± 6.38 h per week, or 3 times a week with average period of 4.93 ± 2.13 h, respectively; average blood flow 293.3 ± 11.6 ml/min).

Treatment modality at the beginning of pUF was mainly CAPD in both groups (HFrEF 50.9% and HFpEF 48.6%). Average Kt/V was 2.62 ± 1.81 in HFrEF and 2.20 ± 0.89 in HFpEF. 10 HFrEF (9.35%) and 3 HFpEF patients (8.12%) required intermittent haemodialysis at different time points after beginning of pUF. 25 HFrEF patients (23.6%) and 11 HFpEF patients (29.7%) received Icodextrin.

Laboratory results are demonstrated in Table 2. The mean within-person change of NTproBNP was more pronounced in HFpEF (− 379 ng/l, relative change − 7%) than in HFrEF (absolute − 2133 ng/l, relative change − 0.5%) patients.

**Table 2:** Laboratory variables at baseline and after beginning of pUF^a^

	Pre-pUF^a^	Post-pUF^a^							
		3 months	***P***	6 months	***P***	12 months	***P***	Last follow- up	***P***
hs TNT^d^ (pg/ml)									
HFrEF^b^	108.0 ± 233.1	77.5 ± 58.7	0.189	136.3 ± 184.7	0.608	105.6 ± 79.5	**0.046**	107.3 ± 39.0	0.949
HFpEF	152.0 ± 236.5	124.3 ± 12.4	0.586	150.0 ± 198.0	0.410	123.3 ± 57.5	0.848	177.5 ± 101.4	0.665
NT proBNP (pg/ml)									
HFrEF^b^	5220.1 ± 4438.4	4944.7 ± 10,351.8	0.404	5242.6 ± 8465.3	0.542	3539.6 ± 5776.2	0.309	6650.0 ± 11,197.5	0.148
HFpEF	2630.3 ± 2029.7	1980.0 ± 2309.6	0.323	2033.9 ± 2701.2	0.645	2059.0 ± 3218.6	0.937	2407.0 ± 5296.6	0.495
Albumin (g/l)									
HFrEF^b^	38.6 ± 5.59	37.8 ± 5.76	0.200	38.0 ± 7.65	0.140	38.9 ± 4.89	0.509	37.9 ± 7.43	0.166
HFpEF	38.0 ± 6.64	35.5 ± 7.73	0.147	36.4 ± 3.91	0.884	39.8 ± 3.27	0.309	35.4 ± 5.54	0.187
Creatinine (mg/dl)									
HFrEF^b^	3.19 ± 3.12	3.14 ± 2.62	0.717	3.49 ± 2.67	0.959	3.30 ± 2.82	0.133	3.90 ± 2.98	0.090
HFpEF^c^	2.65 ± 1.29	2.96 ± 1.62	0.220	3.32 ± 1.97	**0.008**	3.77 ± 2.29	**0.018**	4.00 ± 2.15	**0.008**
MDRD^e^ eGFR^f^ (ml/min/1.73 m^2^)									
HFrEF^b^	23.1 ± 10.6	28.4 ± 18.3	0.240	25.9 ± 14.8	0.351	26.3 ± 14.6	0.620	23.3 ± 19.8	0.841
HFpEF^c^	27.8 ± 13.2	26.5 ± 15.1	0.628	27.4 ± 17.4	0.530	23.3 ± 15.2	0.094	19.5 ± 15.4	**0.042**
BUN (mg/dl)									
HFrEF^b^	145.4 ± 68.0	109.2 ± 84.7	**< 0.001**	103.0 ± 42.5	**< 0.001**	94.1 ± 37.4	**< 0.001**	102.9 ± 42.1	**< 0.001**
HFpEF^c^	153.5 ± 69.2	94.6 ± 32.5	**0.011**	101.4 ± 39.8	**0.015**	105.6 ± 45.4	0.070	117.3 ± 48.8	0.117
CRP (mg/l)									
HFrEF^b^	37.9 ± 59.0	14.4 ± 29.4	**0.002**	9.90 ± 12.5	**0.002**	8.67 ± 8.36	**0.011**	17.3 ± 27.6	**0.009**
HFpEF^c^	19.4 ± 17.6	6.56 ± 7.93	0.067	6.83 ± 8.16	0.069	13.3 ± 26.1	**0.028**	13.7 ± 21.4	**0.019**
Sodium (mmol/l)									
HFrEF^b^	136.2 ± 5.07	137.9 ± 4.88	**0.025**	137.1 ± 4.84	0.127	138.2 ± 4.04	0.055	136.6 ± 5.31	0.992
HFpEF^c^	137.2 ± 3.85	137.4 ± 10.1	0.602	138.3 ± 5.30	0.821	136.9 ± 5.72	0.336	137.1 ± 6.47	0.089
Potassium (mmol/l)									
HFrEF^b^	4.32 ± 0.74	4.22 ± 0.68	**0.035**	4.19 ± 0.60	**0.013**	4.26 ± 0.54	0.213	4.29 ± 0.85	0.217
HFpEF^c^	4.25 ± 0.68	4.14 ± 0.64	0.287	4.50 ± 0.56	0.107	4.02 ± 0.50	0.340	4.36 ± 0.62	0.756
Phosphate (mmol/l)									
HFrEF^b^	1.47 ± 0.51	1.47 ± 0.65	0.984	1.59 ± 0.78	0.771	1.61 ± 0.97	0.315	1.67 ± 1.16	0.146
HFpEF^c^	1.86 ± 1.98	1.72 ± 1.14	0.276	1.54 ± 0.51	0.428	1.37 ± 0.30	0.470	1.86 ± 1.56	0.388
Haemoglobin (mg/dl)									
HFrEF^b^	11.3 ± 1.74	11.9 ± 1.71	**0.024**	11.7 ± 2.20	**0.030**	12.6 ± 1.79	**< 0.001**	11.6 ± 2.09	0.614
HFpEF^c^	10.8 ± 1.68	11.7 ± 2.12	**0.038**	11.9 ± 2.29	**0.014**	11.9 ± 1.87	**0.025**	11.2 ± 2.40	0.105

Data are presented as mean ± SD

^a^pUF (peritoneal ultrafiltration)

^b^HFrEF (heart failure with reduced ejection fraction)

^c^HFrEF (heart failure with preserved ejection fraction)

^d^hsTNT (high sensitive troponin)

^e^MDRD (modification of diet in renal disease)

^f^eGFR (estimated glomerular filtration rate)

Creatinine increased significantly in HFpEF group (2.65 ± 1.29 mg/dl to 4.00 ± 2.15 mg/dl, *P* = 0.008) while MDRD revealed a slight decrease (27.8 ± 13.2 ml/min/1.73 m^2^ to 19.5 ± 15.4 ml/min/1.73 m^2^, *P* = 0.0042). BUN declined more sustained in HFrEF patients (HFrEF 145.4 ± 68.0 mg/dl to 102.9 ± 42.1 mg/dl, *P* <  0.001). BUN/creatinine ratio (BCR) declined significantly in both groups (HFrEF 55.7 ± 21.9 to 34.3 ± 17.9 *P* > 0.001 and HFpEF (50.5 ± 68.9 to 37.6 ± 21.9, *P* = 0.006).

CRP improved in HFrEF and HFpEF with pUF treatment. Albumin remained unchanged over all.

**Fig. 1:**
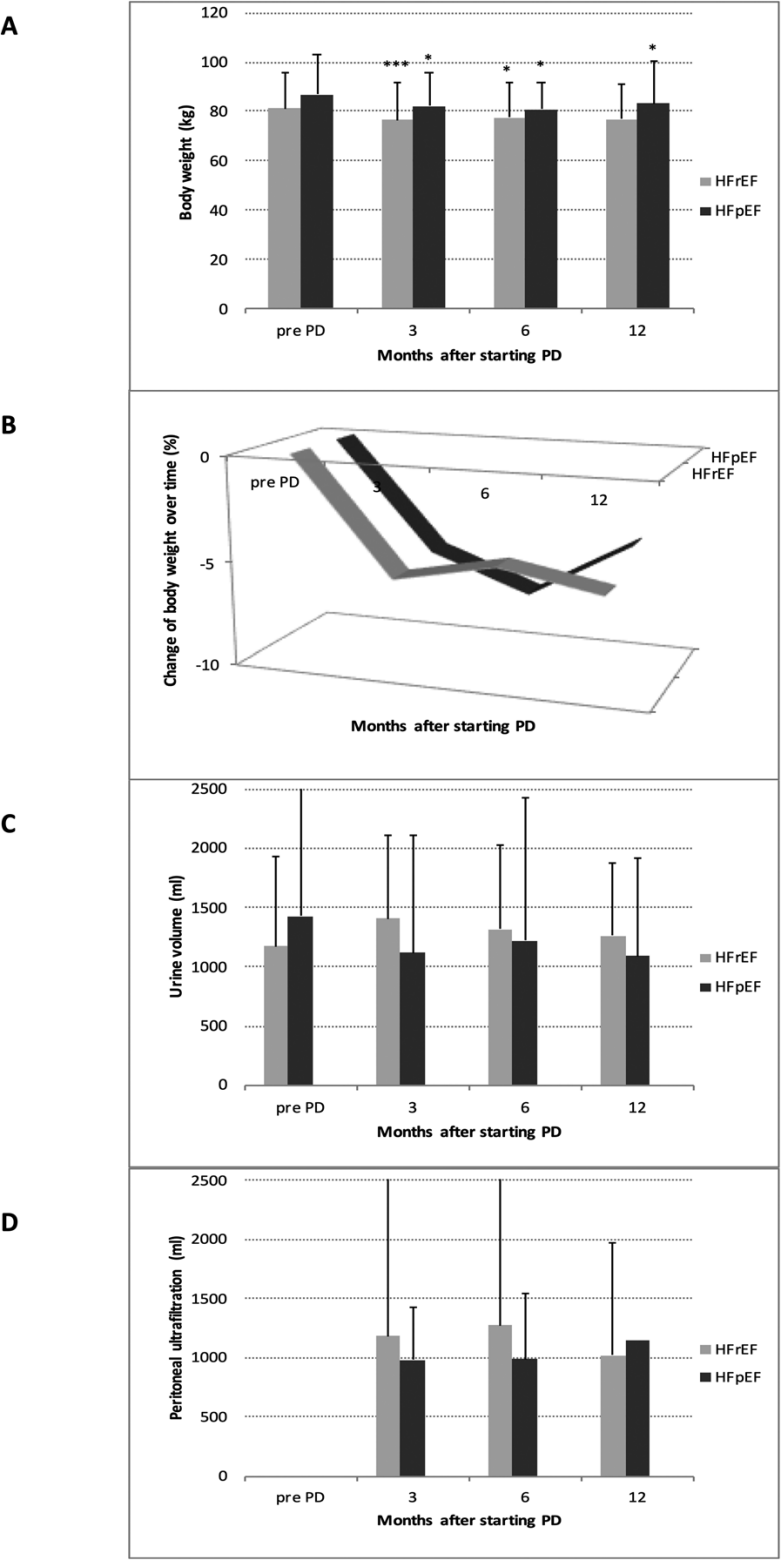
Comparison of body weight (1a), relative change of body weight (1b) urine (1c) and peritoneal ultrafiltration (1d) after starting pUF in HFrEF and HFpEF. Not significant

Follow-up results of clinical variables after beginning of pUF are shown in Table 3. Comparing HFpEF and HFrEF, there were no significant differences regarding pUF and urine volume. Body weight improved in both groups significantly (HFpEF 86.9 ± 15.8 kg to 83.1 ± 15.9, *P* = 0.005 and HFrEF 81.1 ± 14.6 kg to 77.2 ± 15.6, *P* = 0.003) (Fig. 1).

**Table 3:** Clinical variables at baseline and after beginning of pUF^a^. Median, standard deviation and students t-test for paired variables. Mean and Wilcoxon signed-rank text for not normally contributed paired variables

	Pre-pUF^a^	Post-pUF^a^							
		3 months	***P***	6 months	***P***	12 months	***P***	Last follow up	***P***
**NYHA**^d^									
HFrEF^b^	3.45 ± 0.52	2.80 ± 0.50	**< 0.001**	2.79 ± 0.71	**0.001**	2.53 ± 0.75	**< 0.001**	2.71 ± 0.72	**< 0.001**
HFpEF^c^	3.19 ± 0.61	2.82 ± 0.56	**0.002**	2.44 ± 0.68	**0.028**	2.63 ± 0.69	**0.045**	2.72 ± 0.58	**< 0.001**
**Systolic BP**^e^ (mmHg)									
HFrEF^b^	108.4 ± 18.7	111.2 ± 18.9	0.674	111.6 ± 18.9	0.846	117.0 ± 23.1	0.875	108.2 ± 23.2	0.284
HFpEF^c^	115.9 ± 20.7	120.1 ± 17.1	0.703	114.9 ± 20.6	0.680	110.1 ± 19.6	0.354	114.3 ± 22.8	0.799
**Diastolic BP**^e^ (mmHg)									
HFrEF^b^	68.4 ± 12.6	65.8 ± 11.7	**0.001**	67.2 ± 13.3	0.097	69.8 ± 17.0	0.270	66.3 ± 14.2	**0.010**
HFpEF^c^	67.1 ± 10.1	64.7 ± 7.18	0.542	66.5 ± 10.0	0.639	64.2 ± 12.3	0.752	64.0 ± 11.1	0.533
**EF**^f^ (%)									
HFrEF^b^	25.9 ± 6.82	29.0 ± 8.30	0.081	29.6 ± 8.75	0.154	31.1 ± 12.8	0.281	30.4 ± 12.2	**0.046**
HFpEF^c^	51.3 ± 7.77	48.2 ± 5.04	0.304	49.0 ± 4.56	0.404	47.0 ± 5.20	0.547	51.5 ± 8.86	0.189
**Urine** (ml)									
HFrEF^b^	1172.3 ± 759.7	1408.4 ± 697.9	0.199	1319.1 ± 701.3	0.298	1263.4 ± 606.5	0.079	1051.0 ± 676.3	0.637
HFpEF^c^	1431.4 ± 1195.8	1118.3 ± 991.4	0.273	1225.0 ± 1198.9	0.522	1092.3 ± 818.5	0.764	984.6 ± 740.3	0.744
**pUF**^a^ (ml)									
HFrEF^b^	na^g^	1180.6 ± 1391.5	na^g^	1269.6 ± 1566.5	0.805	1020.3 ± 952.1	0.626	1306.8 ± 1284.7	0.870
HFpEF^c^	na^g^	979.7 ± 473.6	na^g^	982.6 ± 442.4	0.335	1140.6 ± 552.6	0.757	997.8 ± 546.6	0.359
**Body Weight** (kg)									
HFrEF^b^	81.1 ± 14.6	76.7 ± 15.2	**< 0.001**	77.4 ± 14.5	**0.002**	76.7 ± 14.3	0.068	77.2 ± 15.6	**0.003**
HFpEF^c^	86.9 ± 15.8	82.3 ± 13.3	**< 0.005**	80.8 ± 11.0	**0.023**	83.2 ± 16.4	**0.046**	83.1 ± 15.9	0.005

Data are presented as mean ± SD

^a^pUF (peritoneal ultrafiltration)

^b^HFrEF (heart failure with reduced ejection fraction)

^c^HFpEF (heart failure with preserved ejection fraction)

^d^NYHA (New York Heart Association functional class)

^e^BP (blood pressure)

^f^EF (ejection fraction)

^g^na (not applicable)

Regarding medication, use of MRA slightly decreased in HFrEF patients (37.4 to 32.0%) and increased in HFpEF patients (35.1 to 47.4%). Use of ACE inhibitors or ARBs decreased (HFrEF 54.2 to 39.7% vs. HFpEF 54.1 to 41.2%) during first year after starting pUF. Meanwhile, use of beta blockers increased during this period (HFrEF 64.5 to 77.6% vs. HFpEF 56.8 to 65.4%).

13 HFrEF patients (12.1%) and 8 HFpEF patients (21.6%) were treated with erythropoiesis stimulation agents (ESA) pre-pUF. This number increased to 18.6% in HFpEF and 37.5% in HFrEF at 12 months. In patients without ESAs or being on stable dosages of ESAs, we initially detected a significant increase of haemoglobin after 3 months (HFrEF from 11.3 ± 1.74 mg/dl to 11.9 ± 1.71 mg/dl, *P* = 0.024 and HFpEF from 10.8 1.68 to 11.7 ± 2.12, *P* = 0.038). Results improved but without significance at the end of the observation period of max. Seventy-two months (HFrEF 11.6 ± 2.09 mg/dl, *P* = 0.614, HFpEF 11.2 ± 2.40 mg/dl, *P* = 0.105).

**Fig. 2:**
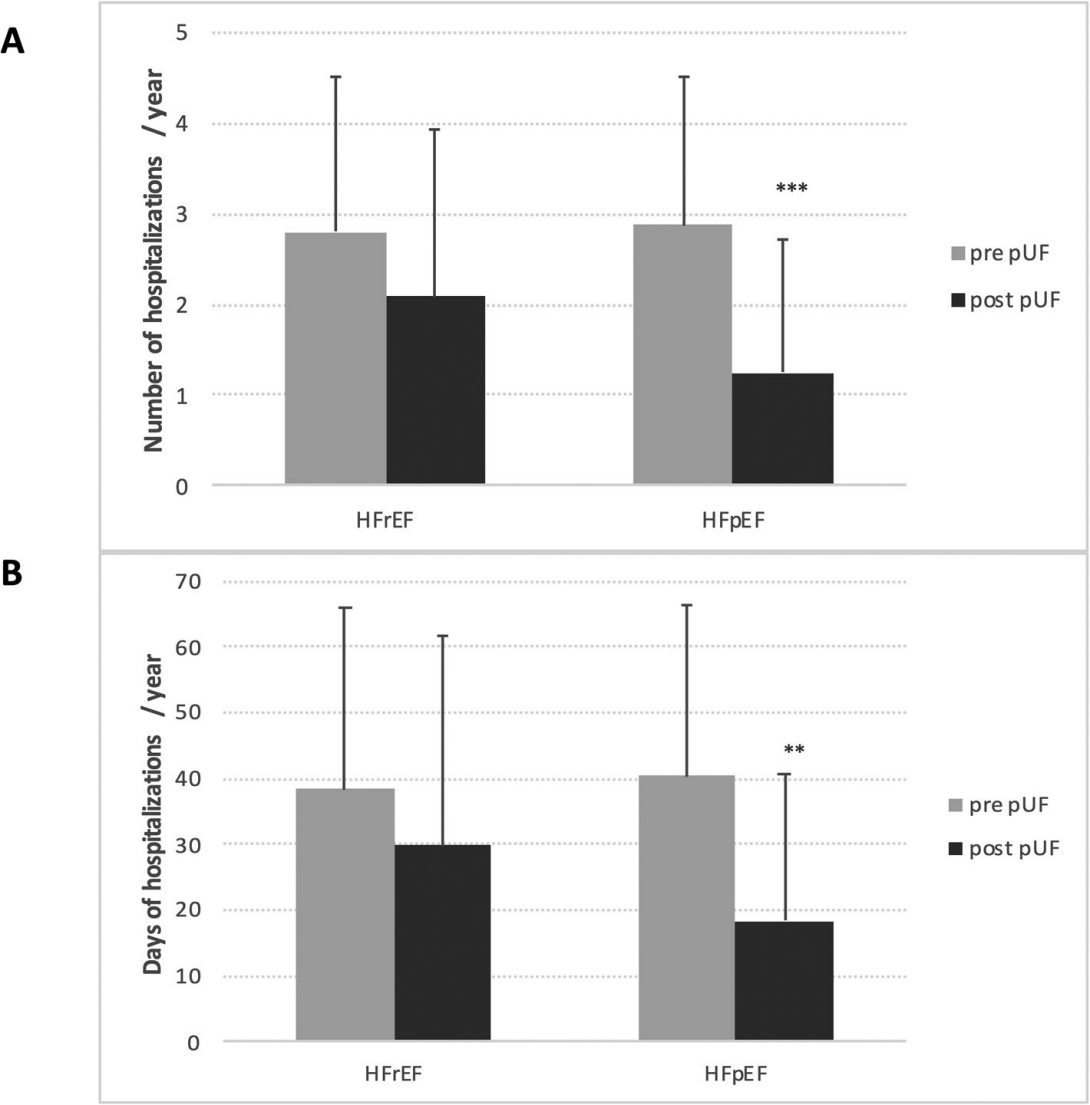
Number of hospitalizations per year after starting pUF for patients with HFrEF and HFpEF (2a) and days of hospitalizations per year after starting pUF for patients with HFrEF and HFpEF (2b). *** *P* <  0.001 and ** *P* <  0.01

Focusing on heart failure entity, 12 months prior to pUF, no differences in number of hospitalizations (*P* = 0.809) and days in hospital (*P* = 0.746) were detected among HFrEF and HFpEF. After initiation of pUF, total number (2.88 ± 1.62 to 1.25 ± 1.45, *P* = 0.001) and days of hospitalizations (40.4 ± 31.7 to 18.3 ± 22.5, *P* = 0.005) significantly decreased in HFpEF, but not in HFrEF patients (2.79 ± 1.70 to 2.09 ± 1.85, *P* = 0.062; 38.5 ± 27.5 to 29.8 ± 25.9, *P* = 0.092, respectively) (Fig. 2a and b).

**Fig. 3:**
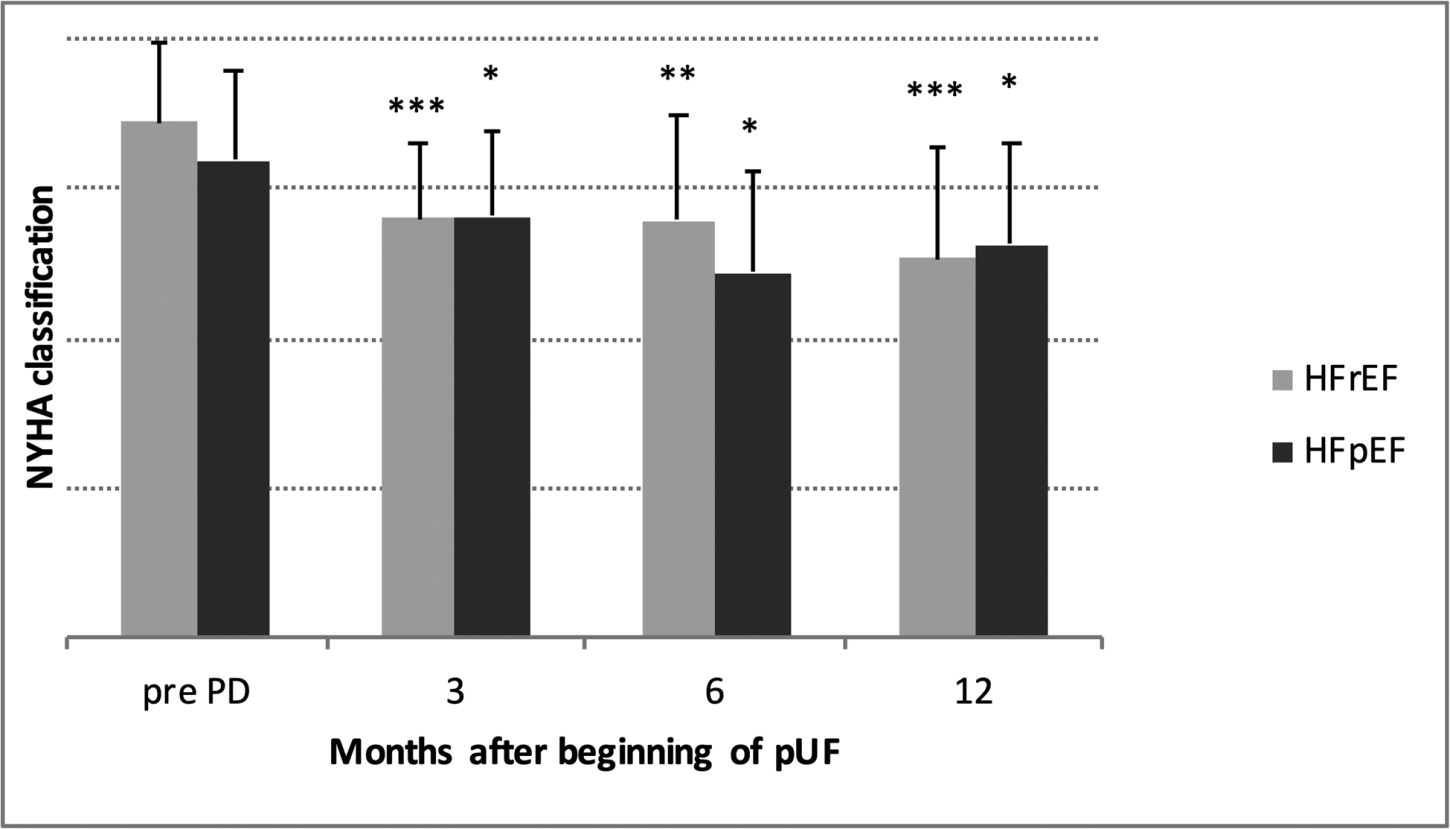
NYHA classification after starting pUF in patients with HFrEF and HFpEF. *** *P* <  0.001, ** *P* <  0.01 and * *P* <  0.05

NYHA improved both in HFrEF (3.45 ± 0.52 to 2.71 ± 0.72, *P* <  0.001) and in HFpEF (3.19 ± 0.61 to 2.72 ± 0.58, *P* <  0.001) (Fig. 3).

HFrEF patients displayed significantly improved LVEF (25.9 ± 6.83% to 30.4 ± 12.2%, *P* = 0.046).

Average time until death was 439.0 ± 471.9 days in HFrEF and 392.9 ± 373.2 days in HFpEF. 4 HFrEF patients recompensated and 1 HFrEF patient received kidney transplantation and therefore intermittently stopped pUF treatment. In addition, 7 HFrEF and 5 HFpEF patients changed medical centres for different reasons, and 38 HFrEF and 12 HFpEF were lost to follow-up. The registry data did not record complications systemically, but in our previous study, we demonstrated that nearly no cardiac decompensations occurred with pUF [32].

**Fig. 4:**
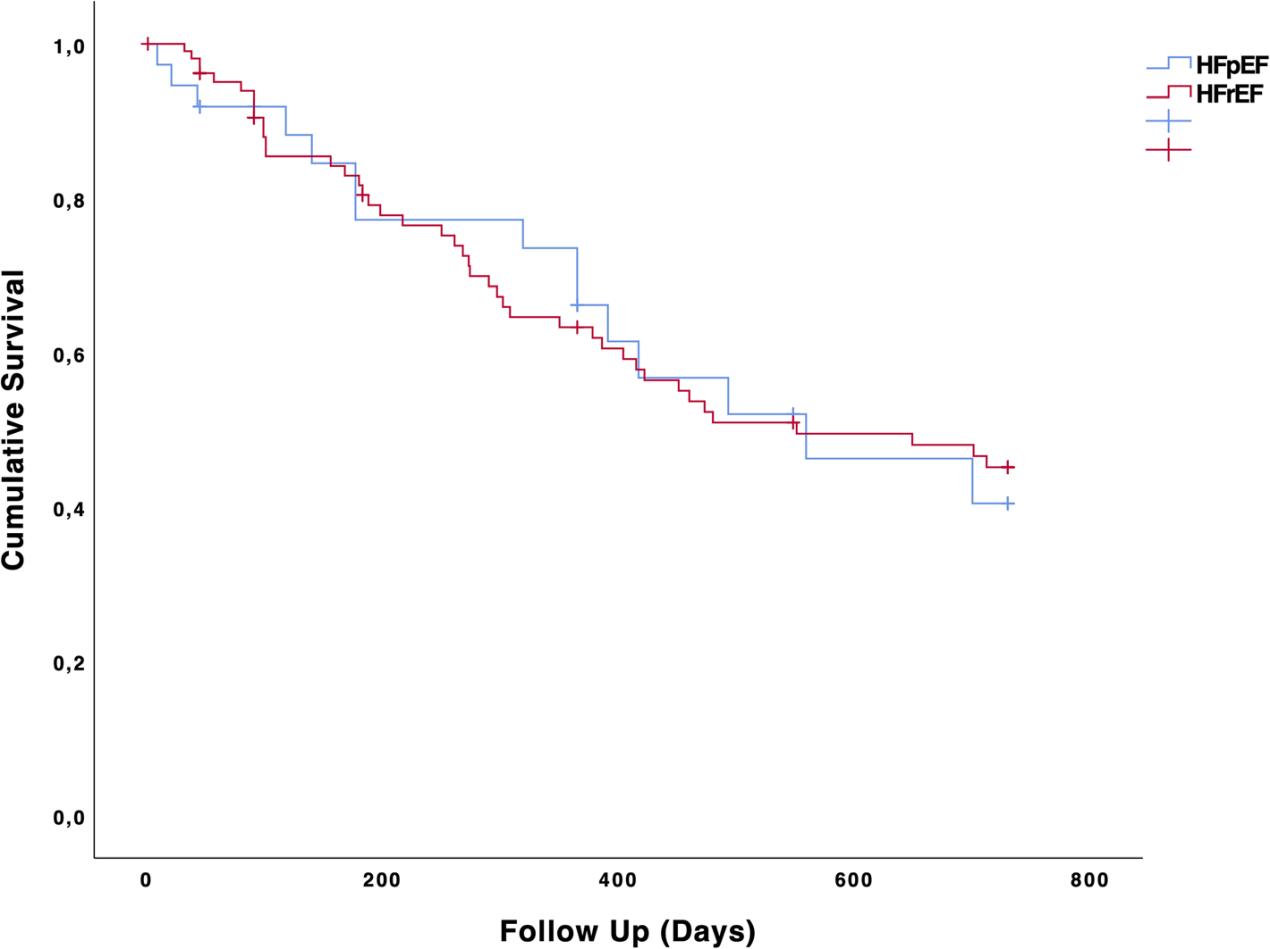
Kaplan-Meier survival curve. Cumulative survival of HFpEF and HFrEF 2 years after starting pUF therapy

There was no significant mortality difference between HFpEF and HFrEF after starting pUF (Fig. 4) (first year *Log-rank* = 0.968) and second year (*Log-rank* = 0.830). Similarly, there was no significant difference in mortality between ICMP and DCMP [33] (first year *Log-rank* = 0.142 and second year *Log-rank* = 0.242 respectively).

## Discussion

This substudy confirms that pUF therapy potentially yields differential outcomes in HFpEF and HFrEF patients. Our findings demonstrate that particularly in HFpEF, but not so in HFrEF patients, pUF significantly reduced both number and days of hospitalization for all causes. The 30-day all-cause readmission rate is still 19% for HF patients [34] and the 1-year hospitalization rate runs up to 43.9% and 31.9%, in HFrEF and HFpEF, respectively. From a medical as well as an economic point of view pUF may prove especially beneficial in HFpEF patients as it contributes to lower healthcare costs by reducing in-hospital days [35, 36]. To date it is unclear what this finding might be related to, but one may speculate that the burden of cardiorenal interaction is even more pronounced in HFpEF as the incidence of pulmonary hypertension and renal dysfunction is slightly higher in this HF subgroup [37, 38].

Various studies on pUF described 1-year mortality rates between 18% and 44% with conventional treatment [39–41]. Wang et al. confirmed an increased mortality of HF patients with pUF treatment, which appeared even more pronounced in case of HFrEF [42]. In our study, however, we did not find any difference regarding 1- and 2 year mortality or between ICMP and DCMP patients, which is most likely related to a highly variable comorbidity load between studies [30].

Our observations correspond with previous results, as NYHA classification improved significantly with pUF in all groups [43]. This symptomatic improvement translated only to significant changes in LVEF in HFrEF patients, which corresponds with findings of Courivaud et al. [41]. On first sight the impact of pUF on change/improvement of EF might depend on baseline EF. In former studies, however, it was demonstrated that LVEF does not add significant prognostic information in patients with advanced CKD [44]. Accordingly, the subgroup analysis of 1- and 2- year mortality did not reveal any difference between DCMP and ICMP patients or between HFpEF and HFrEF patients as far as EF is concerned.

Independent from HF classification, an overall significant weight loss was documented. This finding has to be differentiated, as weight loss may on the one hand be seen as a marker for better volume management due to additional UF with remained urine output. On the other hand weight loss may be regarded as a marker for muscle loss and malnutrition, as PD patients lose several grams of protein per day via the dialysate. In this context it is important to note that albumin levels representing a strong predictor of survival remained within the normal range. We thus conclude that pUF therapy indeed leads to an improved volume management without triggering a significant wasting syndrome. The potential loss of proteins is obviously compensated by an improved resorption of nutritional components when the intraabdominal compartment is decongested as mentioned below.

Interestingly, a low BUN is associated with a significantly improved prognosis in HF patients [45–47]. In our cohort pUF treatment resulted in decreased BUN/creatinine ratios (BCR), more pronounced in HFrEF patients. BCR can be regarded as a more powerful predictor of survival among HF patients with renal dysfunction than conventional renal function measures [48]. But contrary to literature, we observed in patients with HFrEF a lower BCR but no significant decrease in hospitalizations. This conflicting observation might be caused by the more pronounced elimination of BUN with pUF.

Moreover, we found weight loss to be accompanied by significantly lowered CRP and BUN levels in both groups. eGFR slightly decreased especially in HFpEF patients, potentially reflecting a sustained loss of oedema. As interstitial oedema in the intra-abdominal compartment is known to trigger both the translocation of LPS with ensuing secondary inflammation and to impede resorption of nutritional compounds, it can cause profound cachexia [49]. So not only theoretically, pUF allows an intracorporeal, gentle and continuous UF to relieve the reno-venous and intra-abdominal pressure overload while draining ascites and interstitial oedema [50, 51]. This potentially aids to stabilize the remaining glomerular filtration rate and helps to decrease inflammation. These findings might be one of the main advantages as compared to extracorporeal haemofiltration strategies, where rapid intravascular fluid removal causes sympathetic counteractivation with a deterioration of renal function and where intraabdominal congestion is not influenced.

Despite the positive combination of reduced dyspnoea (reflected by improved NYHA classification) and increased weight loss, serial NTproBNP values remained unaffected throughout the study. Looking at the within-person variation instead of the rather large between-person variation [52], however, revealed a different picture: The relative NTproBNP levels decreased in both groups, indicating the positive effects of pUF treatment in CHF, although this effect was more pronounced in HFpEF patients.

Another important aspect is that medical HF therapy in patients with CKD is frequently limited by hyperkalaemia, so patients are less likely to receive effective dosages of ACE inhibitors or ARBs [53]. Interestingly, patients with pUF often display a mild hypokalaemia, which may represent an additional advantage over haemodialysis as it offers the chance to reach a dosage of RAAS blockers or MRAs that would accord to the guidelines [22]. In our patient cohort, initially an adequate medical HF therapy was possible only in 35–54% of patients. Use of ACE/ARBs decreased but use of MRAs increased in HFpEF patients with pUF. Use of beta blockers increased in both groups. Further studies are needed to give clarification about a potential benefit of higher dosage of MRA and beta blockers in this special patient cohort.

Finally, some limitations should be noted. The study comprises a relatively small patient cohort, while cardiorenal patients were included from 18 different centres. This all-comers population may thus have resulted in a highly heterogeneous collective that does not allow the exclusion of potential biases. Heterogeneity, however, is a problem always inherent to the HF syndrome which is driven by the complex situation of multiple comorbidities. In our recent publication [29] we therefore chose to analyse the *Charlson Comorbidity Score* that revealed a halving of the expected mortality. Moreover, the reduced hospital admissions may have been counterbalanced by the complexity of pUF therapy, which should have been controlled for by a standardized quality of life assessment. Our previous study, however, invalidates this objection by demonstrating an improved quality of life with pUF [32]. Finally, the fact that pUF patients received a rather close monitoring might have resulted in an improved outcome on its own.

## Conclusion

To conclude, in our study all congestive HF patients clinically improved. While HFpEF patients might in particular benefit the most with respect to hospitalization, HFrEF patients experienced an improvement concerning EF. This study thus warrants larger controlled studies in order to elaborate the differential effects of pUF as an adjunct palliative therapy in end stage HF. With regard to HFpEF patients it may be especially rewarding to gain more insight concerning the specific cardio-pulmo-renal interactions as this clinical entity still is in search for evidence based therapeutic approach.

## Data Availability

The data that support the findings of this study are available from Gnädig. B. but restrictions apply to the availability of these data, which were used under license for the current study, and so are not publicly available. Data are however available from the authors upon reasonable request and with permission of DGfN and Schwenger V. as well as Remppis A.

## Abbreviations

ACE: Angiotensin converting enzyme
ARB: Angiotensin II receptor blocker
BUN: Blood urea nitrogen
BCR: BUN Creatinine ratio
CAPD: Continuous ambulatory peritoneal dialysis
CKD: Chronic kidney disease
DGfN: Deutsche Gesellschaft für Nephrologie
ESC: European Society of Cardiology
CRP: C Reactive protein
DCMP: Dilated cardiomyopathy
EF: Ejection fraction
eGFR: estimated Glomerular Filtration Rate
HF: Heart failure
HFpEF: Heart failure with preserved ejection fraction
HFrEF: Heart failure with reduced ejection fraction
ICMP: Ischemic cardiomyopathy
LVEF: Left ventricular ejection fraction
MDRD: Modification of diet in renal disease
MRA: Mineralocorticoid receptor antagonist
NYHA: New York Heart Association
PD: Peritoneal dialysis
pUF: peritoneal Ultrafiltration
RAAS: Renin angiotensin aldosterone system
UF: Ultrafiltration
